# Analysis of 17 Prenatal Cases with the Chromosomal 1q21.1 Copy Number Variation

**DOI:** 10.1155/2022/5487452

**Published:** 2022-04-27

**Authors:** Xiaohui Wen, Huanxia Xing, Keyan Qi, Hao Wang, Xiaojun Li, Jianjiang Zhu, Wenqi Chen, Limin Cui, Jing Zhang, Hong Qi

**Affiliations:** ^1^Prenatal Diagnosis Center, Haidian Maternal and Child Health Care Hospital, Beijing, China; ^2^Prenatal Diagnosis Center, Langfang Maternal and Child Health Care Hospital, Langfang, Hebei, China; ^3^Prenatal Diagnosis Center, Beijing Obstetrics and Gynecology Hospital, Capital Medical University, Beijing, China; ^4^Prenatal Diagnosis Center, Hangzhou Women's Hospital, Hangzhou, Zhejiang, China; ^5^Department of Cell Biology and Medical Genetics, School of Medicine, Zhejiang University, Hangzhou, China; ^6^Prenatal Diagnosis Center, Shijiazhuang Obstetrics and Gynecology Hospital, Shijiazhuang, Hebei, China

## Abstract

Copy number variations (CNVs) at the chromosomal 1q21.1 region represent a group of hot-spot recurrent rearrangements in human genome, which have been detected in hundreds of patients with variable clinical manifestations. Yet, report of such CNVs in prenatal scenario was relatively scattered. In this study, 17 prenatal cases involving the 1q21.1 microdeletion or duplication were recruited. The clinical survey and imaging examination were performed; and genetic detection with karyotyping and CNV analysis using chromosomal microarray (CMA) or CNVseq were subsequently carried out. These cases were all positive with 1q21.1 CNV, yet presented with exceedingly various clinical and utrasonographic indications. Among them, 12 pregnancies carried 1q21.1 deletions, while the other 5 carried 1q21.1 duplications, all of which were within the previously defined breaking point (BP) regions. According to the verification results, 9 CNVs were *de novo*, 7 were familial, and the other 1 was not certain. We summarized the clinical information of these cases, and the size and distribution of CNVs, and attempted to analyze the association between these two aspects. The findings in our study may provide important basis for the prenatal diagnosis and genetic counseling on such conditions in the future.

## 1. Introduction

The 1q21.1 copy number variations (CNVs) represent a group of recurrent deletions and duplications at the subchromosomal region 1q21.1q21.2 (GRCh37/hg19, chr1:144.0–149.5 Mb) [1], which have been reported to be associated with a spectrum of diverse clinical features [2–4]. In this region, there are multiple low copy repeats (LCR) clustering into four main segmental duplication blocks with a size ranging from ~270 kb to 2.2 Mb (BP1-BP4; BP representing break point), of which the nonallelic homologous recombination (NAHR) has been considered as the major mechanism of these CNVs [2, 3, 5, 6].

According to the Online Mendelian Inheritance in Man database (OMIM, https://www.omim.org/), symptomatic individuals with these 1q21.1 CNVs are classified into three main syndromes: 1q21.1 deletion syndrome (MIM #612474), 1q21.1 duplication syndrome (MIM #612475), and TAR (thrombocytopenia-absent radius) syndrome (MIM #274000). These syndromes share certain clinical phenotypes such as global developmental delay (GDD), craniofacial abnormalities (microcephaly or macrocephaly), and cardiac anomalies [1, 7, 8]. Other mental or psychiatry phenotypes include intellectual disability (ID), autism spectrum disorder, and schizophrenia [9, 10]. Based on the CNV sizes and locations, these deletions and duplications were grouped into 2 classes: class I del/dup, occurring at the 1q21.1 distal region between BP3 and BP4 with a size range of 800 kb–2 Mb; class II del/dup, commonly residing between BP1/BP2 and BP4 with bigger sizes of ~3 Mb or larger [1, 2, 7]. In terms of TAR syndrome, its characteristic features include bilateral absence of radial bones and thrombocytopenia (<50 platelets/nl) [11]. It has been elucidated that the mechanism of TAR syndrome is the biallelic variations in the *RBM8A* gene (MIM ∗ 605313, Chr1: 145,921,555-145,927,483) [12], typically including the situation of a deletion at the proximal 1q21.1 region (a.k.a., TAR deletion) with a minimal size of 200 kb that encompasses the *RBM8A* gene combining with a point mutation in the noncoding region of *RBM8A* [13]. Contrastingly, the impact of duplications of this region is not quite explicit, although one report indicated it to be responsible for certain features like short stature and feeding problems [10].

The fetal indication of subjects with 1q21.1 CNVs has attracted extensive attention in the prenatal diagnosis field [14–16]. However, the detection and prognosis evaluations on these CNVs are still challenging, mainly because of their unclear presentation during pregnancy and variable phenotype. In previous studies, clinicians made the diagnosis mainly counting on genetic copy number analysis triggered by imaging findings of cardiac and neurodevelopmental abnormalities [17–20], which may have certain limitation resulting in missed diagnosis [7].

In the present study, 17 cases involving 1q21.1 CNVs (by microarray or sequencing assay) were enrolled out of a four-year multicenter cohort. We summarized their clinical manifestations and the location and size of the genetic alterations, in order to provide a basis for analyzing the prenatal genotype-phenotype association of this condition.

## 2. Materials and Methods

### 2.1. Subjects

This study was approved by the ethical committee of the Haidian Maternal and Child Health Hospital. Informed consent was signed by the participants, in compliance with the Declaration of Helsinki. Seventeen prenatal cases were recruited in the 4 prenatal diagnosis centers (institution 1, 2, 4, and 5 in the title page), between January 2017 and November 2021. Clinical information was collected, and prenatal ultrasound diagnosis was conducted. Amniocentesis was performed to obtain the fetal samples for subsequent karyotyping and CNV analysis. Parental peripheral blood samples were also collected for further testing.

### 2.2. Karyotyping and Chromosomal Microarray Analysis (CMA)

Conventional G-band karyotyping was performed on prenatal specimens of the fetuses according to the AGT Cytogenetics Laboratory Manual, 4th Edition [21]. The metaphase cells were analyzed according to the 2016 edition of the International System for Human Cytogenomic Nomenclature (ISCN 2016) (https://international-sustainable-campus-network.org/).

Genomic DNA from prenatal amniotic fluid samples of the participants was extracted using the QIAamp DNA mini kit (Qiagen, DEU), and the DNA samples were measured using Nanodrop 2000 (Thermo, USA) for quality control and concentration. Chromosomal microarray analysis (CMA) was performed using Affymetrix Cytoscan HD platform, which comprised of both copy number and single-nucleotide polymorphism (SNP) probes within a whole-genome array (Affymetrix, USA). The specimens were prepared following the manufacturer's standard operating procedures. The data were analyzed using ChAS software, a code analysis suite.

### 2.3. CNVseq by Low Coverage Whole-Genome Sequencing

Copy number variation sequencing (CNVseq) was used to detect chromosome abnormality at low-coverage whole-genome sequencing level. Briefly, the quality of genomic DNA was verified by two methods in combination: (1) DNA degradation and contamination were monitored on 1% agarose gels; (2) DNA concentration was measured by Qubit® DNA Assay Kit in Qubit® 2.0 Flurometer (Life Technologies, CA, USA). Then, sequencing library was generated using CLEANNGS DNA kit following manufacturer's recommendations, and index codes were added to each sample. The clustering of the index-coded samples was performed on a cBot Cluster Generation System using Novaseq5000/6000 S4 Reagent Kit (Illumina) according to the manufacturer's instructions. After cluster generation, the DNA libraries were sequenced on Illumina NovaSeq 6000 platform, and 150 bp paired-end reads were generated. Raw data of fastq format were firstly processed through primary quality control. Clean reads were compared with reference human genome (UCSC hg19) by using BWA software, and the results were converted into bam format and sorted by samtools software. Finally, basic information statistics and map comparison statistics were conducted. CNVnator and BIC-Seq were utilized to do CNV detection. Enliven® was performed to do annotation for CNVs. Enliven® and ANNOVAR [22] were performed to do annotation for VCF (Variant Call Format). Database of Genomic Variants, DECIPHER database, ClinVar, OMIM, and ClinGen were used for interpretation and classification of the clinical significance of candidate CNVs according to previously reported guidelines [23].

## 3. Results

### 3.1. Clinical Manifestations

The major fetal and parental clinical indications were summarized in Table 1. The average age of the 17 gravidae was 28.4 (20-39), with no obvious tendency to advanced age.

**Figure 1:**
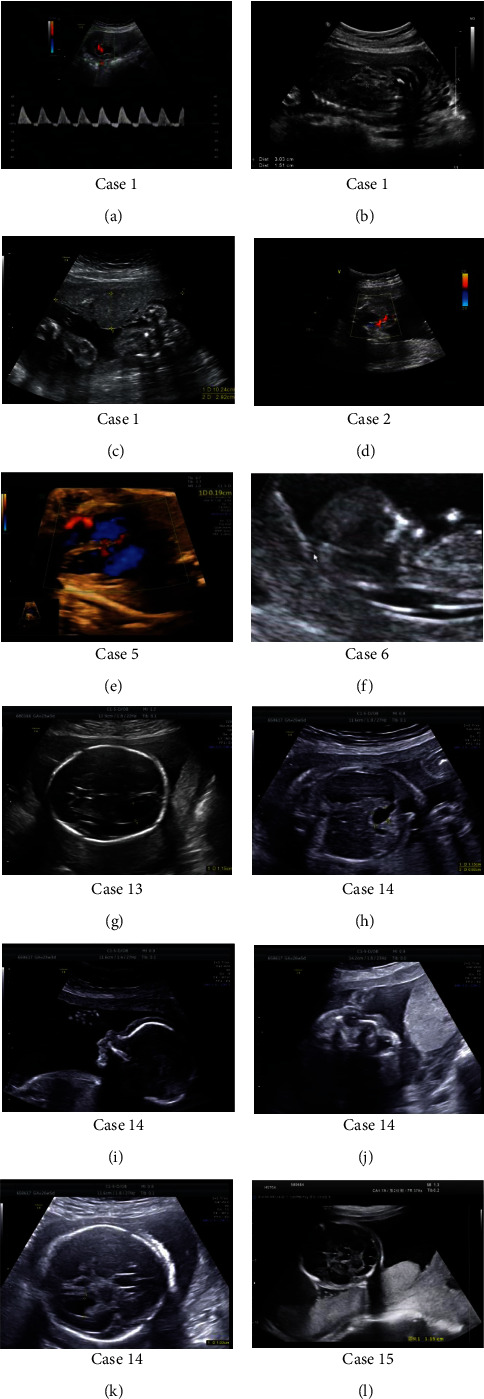
Representative prenatal ultrasonographic indications in these cases. Case 1: (a) umbilical artery blood flow spectrum in diastolic phase disappeared and reversed; (b) increased intestinal echo; (c) small placenta. Case 2: (d) single umbilical artery. Case 5: (e) ventricular septal defect. Case 6: (f) nuchal translucency thickening. Case 13: (g) dilated left lateral ventricle. Case 14: (h) duodenal stricture; (i) micrognathia; (j) wide eye distance; (k) “Strawberry head,” dilated lateral ventricles. Case 15: (l) cerebellar medulla pond widening.

Ten cases manifested mild to moderate prenatal ultrasonographic indications at different gestational weeks, mostly in cardiac and cerebral development (cases 1, 2, 5, 6, 10, 11, and 13-16; with the proportion of ~59%, 10/17). The representative ultrasonographic manifestations were displayed in Figure 1. For the other 7 cases (cases 3, 4, 7-9, 12, and 17), prenatal diagnosis (PD) was carried out not based on ultrasound indications but on other causes; interestingly, there was no obvious abnormity on ultrasonography in any of the 7 cases according to the subsequent examinations. In these 7 cases, the PD reasons of 3 were based on the high risk results of the serological screening (Trisomy 21); three were based on the hints of noninvasive prenatal testing (NIPT) results; the other 1 was because the gravida had history of congenital ventricular septal defect (Table 1).

The follow-up so far revealed that 7 pregnancies were selectively aborted, and the other 10 were continued (see Table 1). Among these 10 remained pregnancies, 3 full-term delivered infants had normal development in their early stage (cases 3, 4, and 15); and follow-up of the others is still ongoing. Additionally, combined with the genetic findings, we particularly focused on the phenotypic features of the carrier parents of inherited CNVs in cases 2, 4, 8, 9, 11, 13, and 16. Two parents had phenotypes to a certain extent: the father in case 4 exhibiting microcephaly, and the mother in case 8 having congenital heart disease. And the other 5 parents were asymptomatic.

### 3.2. Genetic Findings

**Figure 2:**
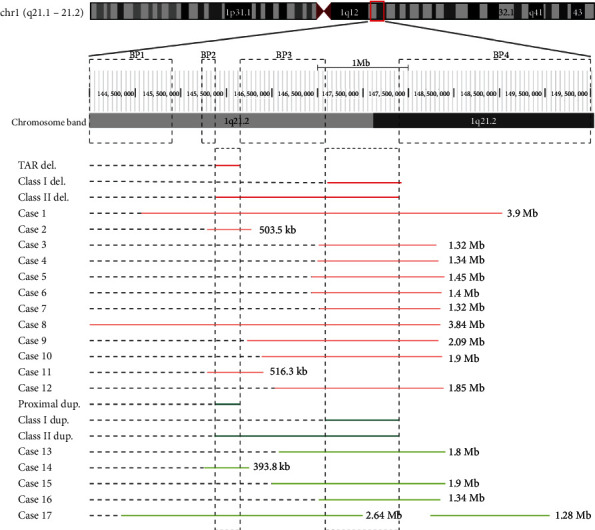
The schematic of all 17 CNVs at 1q21.1 (GRCh37/hg19, 144.0–149.5 Mb) region in this study. Red or orange bars, deletions; green bars, duplications; breakpoint regions (BP1-BP4) in dotted blocks. This model diagram refers to Pang et al., 2020 [1].

According to the conventional G-banding assay, the karyotypes of all 17 prenatal subjects were normal. CMA or CNVseq results identified positive CNVs of 1q21.1 deletions or duplications in all cases, which were depicted in Figure 2 and detailedly listed in Table 1. Twelve cases were with 1q21.1 deletions, and the other 5 were with 1q 21.1 duplications. Nine of these CNVs were *de novo* in their respective pedigrees, 7 were inherited, and the other 1 (case 3) was not certain.

In the 12 cases with segmental deletions, the minimum fragment length is 503.5 kb (case 2) and the maximum is 3.9 Mb (case 1). In the 5 cases with segmental duplications, the minimum fragment length is 393.8 kb (Case 13), and the maximum is 3.92 Mb (case 17). The breaking points of all CNVs fall within the four BP blocks described previously, making them consistent with TAR deletion, class I/II deletion, proximal duplication, or class I/II duplication (details in Figure 2).

## 4. Discussion

In the past 20 years, copy number variation (CNV) has attracted much concentration in the prenatal diagnosis field for genetic disorders or spontaneous miscarriage, while a series of CNV detection techniques have been widely used accordingly [24–28]. The 1q21.1 CNVs initially came to researchers' attention in large cohorts of patients with intellectual disability [29, 30]. Klopocki et al. then clarified the relationship between the biallelic complex variation in this region and thrombocytopenia-absent radius (TAR) syndrome [12], and Brunetti-Pierri et al. systematically classified the recurrent CNVs in this region by summarizing the clinical indications and CNV size and location information of 36 cases [2]. In general, the frequency of the 1q21.1 CNVs is over 0.2% of individuals with developmental delays, intellectual disabilities, and/or congenital anomalies evaluated by CMA [2, 3]. In the prenatal scenario, there has been a lack of large cohorts with 1q21.1 CNVs, which makes it difficult to analyze their impact on *in utero* development [14, 18, 20, 31–33]. To our best knowledge, the present study aggregated a prenatal cohort with the largest sample size. According to our rough calculation, the prenatal incidence of 1q21.1 CNVs is approximately 0.1% (17/18,992; note: the total was derived from the sum of prenatal diagnoses at the institutions involved in the study).

In this study, CNV analysis with CMA or CNVseq approach identified 12 microdeletions and five microduplications at the 1q21.1 region. To be specific, 11 deletions/duplications were located at the BP3-BP4 segment of 1q21.1 region, 3 covered the major part from BP1 to BP4, and the other 3 covered the TAR deletion (or proximal duplication) region. For the 2 class II deletions (case 1 and case 8), they appeared to have a higher risk of fetal abnormalities. Although no structural abnormalities were detected in the fetus in case 8, we should not ignore the fact that the mother had a congenital ventricular septal defect, so attention should be paid in future examinations. For the 2 TAR deletions (case 2 and case 11), follow-up of the fetuses showed that there was no problem in their development, especially no TAR, so we assumed that the two fetuses did not have mutations in the other allele of *RBM8A*. In terms of the 8 class I deletions, we did not find out the pattern of them leading to prenatal indications, but the actual result is that half cases chose abortions depending on specific circumstances. For the 5 microdupulication cases, it is difficult to generalize a pattern of pathogenicity related to the CNV size and distribution based on current clinical and genetic data. Therefore, for clinical consultation, it is necessary to make a comprehensive judgment combined with specific prenatal indications. Case 14 is the only one of 1q21.1 proximal duplication in our study, in which the fetus presented with severe intracranial symptoms. The mechanism and etiology of this duplication are still pending, and further investigation is needed [34, 35].

It was reported that 18% ~50% of 1q21.1 CNVs were *de novo* [7]. Little information is available regarding penetrance of the 1q21.1 recurrent CNVs; similar to several other recurrent microdeletions (e.g., 16p11.2 and 15q13.3), the 1q21.1 CNVs could be inherited from a parent with minimally abnormal or completely normal clinical findings [2, 3, 7, 11]. Based on our current data, it is arbitrary to conclude that *de novo* CNVs is more likely to cause prenatal symptoms than those hereditary, which needs to be clarified by larger sample sizes. Yet, we noticed that families with *de novo* CNVs were more likely to end their pregnancies, while families with hereditary CNVs were more likely to continue (Table 1). Familial CNVs conform to the autosomal dominant pattern, and the risk of recurrence is 50%; the recurrence risk of sporadic CNVs is less than 1%, but higher than in the general population due to the possibility of gonadal mosaicism, which should be noted in counseling for affected families [1, 20, 36]. Moreover, in respect to cases 4 and 9, although the variants were determined as pathogenic at genetic level, the paternal carriers showed no or nearly no symptoms, again reconfirming the ambiguity of the disease penetrance. In addition, we found that in 3 of our cases, serological screening during early pregnancy suggested a high risk of T21, which had not been previously reported. Whether there is a correlation between 1q21.1 CNVs and serological indicators needs to be explored in further experiments. From the perspective of clinical imaging, due to the limitations of intrauterine ultrasound detection and incomplete development of fetus, we cannot judge the possible indications such as abnormal facial or toe features [18].

The main limitation of this study is that although we recognized that the main prenatal indications for 1q21.1 CNVs could be cardiac and cerebral developmental malformations, we still cannot establish a refined genotype-phenotype association. Given that the incidence of 1q21.1 CNVs is almost comparable to that of classic microdeletion disorders such as Di George syndrome, we call for further studies with larger cohort sizes.

In summary, we provide the largest cohort to date of prenatal cases involving 1q21.1 CNVs. For these variations with exceeding clinical heterogeneity, our findings are helpful to further explore their prenatal pathogenicity pattern. At the same time, our study may also benefit clinicians in providing better counseling when it comes to these variations.

## Figures and Tables

**Table 1:** Clinical characteristics and genetic variations of the seventeen cases.

Case no.	Maternal age (Yr)	No. of gravida and para; gestational week with diagnosis∗	Ultrasonic diagnostic indications	Clinical follow-up	Karyotype	CNV information	Origin (indications of parent)	Pathogenicity rating∗
1	29	G1P0; 21W2D	Fetal intestinal echo enhanced; high value of fetal umbilical artery blood flow spectrum S/D (the diastolic phase of blood flow spectrum disappeared or reversed occasionally); low value of fetal ultrasound measurement; low volume of placenta size	Aborted at 23W5D	46,XX	arr[hg19]1q21.1q21.2(144, 603, 950 − 148,520,164) × 1 (size: 3.9 Mb)	*De novo*	P
2	27	G2P1; 30 W	Single umbilical artery; hydramnios	Cesarean section; normal development	46,XX	arr[hg19] 1q21.1(145, 382, 123 − 145,885,646) × 1 (size: 503.5 kb)	Maternal (asymptomatic)	VUS
3	20	G1P1; 17W2D (following the hint by NIPT)	No abnormality identified	Full term delivered; normal till now (3 months)	46,XY	seq[hg19]1q21.1q21.2(146,500,001-147,820,000)del (size: 1.32 Mb)	Unidentified	P
4	33	G1P1; 18 W1D (T21 high risk by serological test)	No abnormality identified	Full term delivered; normal till now (10 months)	46,XX	arr[hg19]1q21.1q21.2(146, 488, 130 − 147,830,830) × 1 (size: 1.34 Mb)	Paternal (microcephaly, normal intelligence)	P
5	30	G1P0; 25W4D	Ventricular septal defect; racket placenta	Aborted at 28 W	46,XY	seq[hg19]1q21.1q21.2(146,460,001-147,910,000)del (size: 1.45 Mb)	*De novo*	P
6	35	G3P2; 19W1D	Nuchal translucency thickness (0.45 cm)	Aborted at 22W3D	46,XX	seq[hg19]1q21.1q21.2(146,460,001-147,860,000)del (size: 1.4 Mb)	*De novo*	P
7	31	G2P1; 17W6D (T21 high risk by serological test)	No abnormality identified	Aborted at 21W5D	46,XX	seq[hg19]1q21.1q21.2(146,520,001-147,840,000)del (size: 1.32 Mb)	*De novo*	P
8	21	G1P0; 17W6D (amniocentesis performed owing to maternal abnormality)	No abnormality identified	No abnormality at 22 W of gestation; follow-up continued.	46,XY	seq[hg19]1q21.1q21.2(144,000,001-147,840,000)del (size: 3.84 Mb)	Maternal (congenital ventricular septal defect)	P
9	27	G3P1; 18 W2D (following the hint by NIPT)	No abnormality identified	Follow-up continued	46,XX	arr[hg19]1q21.1q21.2(145, 747, 846 − 147,830,830) × 1 (size: 2.09 Mb)	Paternal (asymptomatic)	P
10	20	G1P0; 22W5D	Narrow septum pellucidum; left lateral ventricle dysplasia	Aborted at 26W5D	46,XX	arr[hg19]1q21.1q21.2(145, 895, 747 − 147,885,600) × 1 (size: 1.9 Mb)	*De novo*	P
11	26	G2P1; 18 W	Nuchal translucency thickness (0.31 cm)	No abnormality at 22 W of gestation; follow-up continued.	46,XY	arr[hg19] 1q21.1(145, 372, 552 − 145, 888, 926) × 1 (size: 516.3 kb)	Maternal (asymptomatic)	VUS
12	34	G1P0; 20W3D (following the hint by NIPT)	No abnormality identified till	Follow-up continuing.	46,XX	seq[hg19]1(q21.1q21.2(146,053,252-147,898,839)del (size: 1.85 Mb)	*De novo*	P
13	39	G2P1; 24W3D	Left lateral ventricle slightly widening	Normal development	46,XY	arr[hg19]1q21.1q21.2(146, 096, 700 − 147,933,973) × 3 (size: 1.8 Mb)	Paternal (asymptomatic)	P
14	33	G4P1; 23W2D	Cranial and facial abnormalities (strawberry head, micrognathia); bilateral lateral ventricle widening; distal duodenum stenosis; MRI: poor formation of cerebral sulcus; several brain measurements below normal	Aborted at 28 W4D	46,XY	arr[hg19]1q21.1(145, 382, 123 − 145,775,966) × 3 (size: 393.8 kb)	*De novo*	VUS
15	22	G1P0; 23W5D	Cerebellar medulla pond widening; MRI: ependymal cyst?	Full term delivered; normal till now (5 months)	46,XX	arr[hg19]1q21.1q21.2(146, 023, 923 − 147,933,973) × 3 (size: 1.9 Mb)	*De novo*	P
16	29	G1P0; 27W3D	Left side hydrocephalus, right lateral ventricle widening	Aborted at 29 W	46,XY	seq[hg19]1q21.1q21.2(146,500,001-147,840,000)dup (size: 1.34 Mb)	Paternal (asymptomatic)	P
17	30	G2P1; 14W3D (T21 high risk by serological test)	No abnormality identified till birth	Full term delivered; lost follow-up	46,XX	arr[hg19]1q21.1(144, 355, 396 − 146,999,427) × 3 (size: 2.64 Mb), 1q21.2(147, 753, 014 − 149,034,959) × 3 (size: 1.28 Mb)	*De novo*	VUS, VUS

∗G: gravida; P: para; W: week; D: day; NIPT: noninvasive prenatal testing; P: pathogenic; B: benign; VUS: variation with unknown significance (rating by the general criteria presented in Ref. [23]).

## Data Availability

The data used to support the findings of this study are included within the article.
